# Supplementary material for: Factors Influencing the Adoption of Magnetic Resonance-Guided High-Intensity Focused Ultrasound for Painful Bone Metastases in Europe, A Group Concept Mapping Study
Source: Int J Environ Res Public Health. 2023 Jan 7;20(2):1084. doi: 10.3390/ijerph20021084 (PMC9858703; doi:10.3390/ijerph20021084)
Supplement: Supplementary file 1 [file ijerph-20-01084-s001.zip › ijerph-2060160-supplementary.pdf]

Suplemmentary Material

Table of contents

File S1. Informed Consent and participant questions 2

File S2. Calculation of the stress value 4

File S3. Editing statements 5

File S4. Point Map........ 7

File S5. Statements per Cluster 8

File S6. Statements located in the northeast quadrant of the Go-Zone........ 10

File S1. Informed Consent and Participant Questions

You have been invited to participate in a web-based participatory research, which is part of the FURTHER Project - a Horizon 2020 Project, Grant Number 825859.

By accepting this informed consent, you acknowledge that your participation is voluntary and that the results of this project may be published in an aggregate manner.

For this project, participants will be asked to contribute to the following phases: (i) Brainstorming with the group based on the focus prompt; (ii) Rating and sorting the ideas generated by the group. You will also be asked to provide non-identifying information about yourself. Your participation is confidential and your input will be made available to other participants anonymously.

You will be reminded to come back to our platform when inputs from other participants are available and when there are new activities open for you. You may participate in the entire project or in one of the two phases.

☐ ACCEPT ☐ DECLINE

Table S1. Demographic questions

	Description	Question	Closed Answers
**1**	FURTHER Consortium	Are you already a member of the FURTHER Consortium?	• Yes<br>• No
**2**	Country	In which country are you based?	• The Netherlands<br>• Germany<br>• Italy<br>• Finland<br>• Other
**3**	Expertise	How would you describe your expertise in relation to MR-HIFU provision?	• **Patient**, caretaker or patient representative<br>• **Expertise on performing HIFU treatment** (e.g. radiologist, interventional radiologist)<br>• **Expertise on other medical specialties** (e.g. radiation oncologist, radiotherapist, oncologist, gynecologist, orthopedic surgeon, anesthesiologist)<br>• **Expertise on the HIFU technology** (e.g. research scientist on experimental imaging, clinical researcher, physicist)<br>• **Expertise on the Value Proposition/ Financial aspects** (e.g. representative for a regulatory agency, or Health Technology Assessment agency, hospital manager, technology provider or developer, market access, entrepreneurial service organization)
**4**	Educational background	What is your educational/academic background?	• Radiology<br>• Radiation Oncology or Radiotherapy<br>• Oncology<br>• Orthopedic Surgery<br>• Anesthesiology<br>• Biological Engineering<br>• Physics<br>• Health Economics<br>• Business / Public administration<br>• Epidemiology<br>• Other
**5**	Prior Knowledge	How would you rate your knowledge on the latest evidence on MR-HIFU for cancer induced bone pain?	• Excellent<br>• Good<br>• Regular<br>• Low<br>• None

File S2. Calculation of the Stress Value

Stress is calculated as:

$$S = \sqrt{\frac{\sum_{r,s}(d_{rs} - \hat{d}_{rs})^2}{\sum_{rs} d^2{}_{rs}}}$$

Where:

d_{rs} is the distance between two points (r and s) in input data matrix

$\hat{d}_{rs}$ is the distance between two points (r and s) in the final point map

File S3. Editing Statements

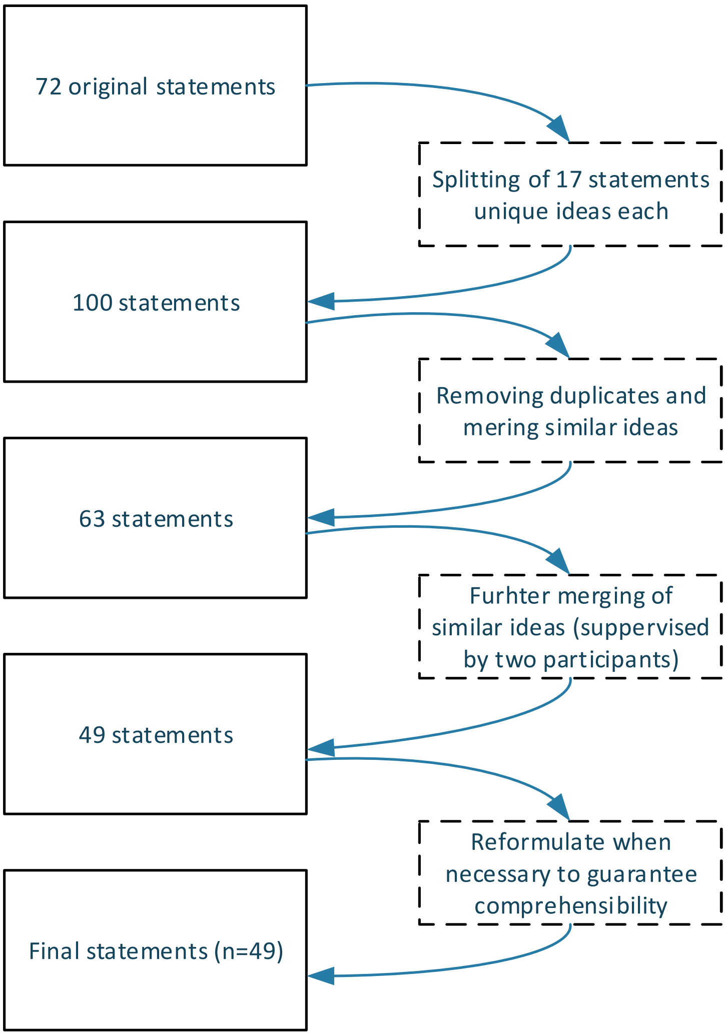

Figure S1. Flowchart the process of splitting and merging statements

Table S2. Examples of splitting and merging of statements

Original statement(s)	Adjusted statement(s)
**Splitting**	
• Difficult patient recruitment, due to the large range in referring medical specialists and their unfamiliarity with HIFU	• Difficult patient recruitment, due to the large range in referring medical specialists<br>• Unfamiliarity of referring physicians with HIFU
**Removing duplicates**	
• Lack of reimbursement<br>• Missing reimbursement is an issue in many countries making the	• Reimbursement of MR-HIFU as inpatient procedure<br>• Reimbursement of MR-HIFU as outpatient procedure

adoption of MR-HIFU difficult or impossible<br>• Reimbursement inside the hospital is essential<br>• Reimbursement in ambulatory care is essential	
**Comprehensibility**	
• organizing GA in a radiology suite (where the MRI is) is an extra cost for the equipment (can be solved once and for all)	• High additional costs related to general anesthesia [removed abbreviation, shortened the statement]

File S4. Point Map

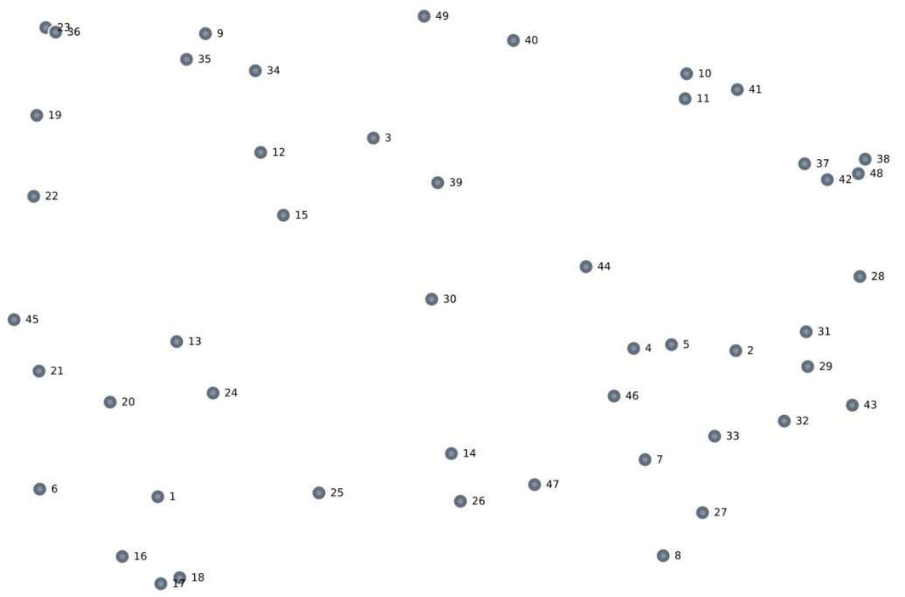

Figure S2. Point Map: statements (and respective identification numbers) plotted on an x–y chart.

File S5. Statements per Cluster

Table S3. Statements per cluster and average perceived importance.

Cluster			Average of perceived importance of each statement					Coherence of perception among countries
	ID	Statement	IT	NL	DE	FI	All participants	
(1)	**Competitive treatments**		**1,29**	**1,36**	**1,71**	**1,07**	**1,36**	**0,07**
	1	Conflict of interest from referring physicians favoring other treatment alternatives	2,00	2,50	3,00	1,67	2,29	0,34
	6	Availability of ultrasound-guided HIFU as a competitive treatment alternative	1,18	2,00	2,00	2,00	1,72	0,17
	16	Availability of cryotherapy as a competitive treatment alternative	0,73	0,86	1,29	0,50	0,84	0,11
	17	Availability of electro chemotherapy as a competitive treatment alternative	1,18	0,71	1,14	0,50	0,94	0,11
	18	Availability of embolization as a competitive treatment alternative	1,36	0,71	1,14	0,67	1,03	0,11
(2)	**Physicians' attitude**		**2,44**	**2,58**	**2,50**	**2,25**	**2,45**	**0,02**
	13	Unfamiliarity/ lack of knowledge among referring physicians with MR-HIFU as a treatment option	2,55	3,11	3,29	3,17	2,97	0,11
	20	Risk avoidance of the medical profession (preference for staying with established treatment methods)	2,18	2,63	2,57	2,00	2,34	0,09
	21	Intrinsic inertia to new treatments (due to overload of the medical profession)	2,18	2,00	2,14	1,67	2,03	0,05
	24	Limited indication for only a small subgroup of bone metastasis patients	2,82	2,89	2,43	2,50	2,70	0,05
	45	Bone metastases patients are often unfit for general anesthesia	2,45	1,88	2,00	2,33	2,19	0,07
	14	Difficult patient recruitment, due to large range in referring medical specialists	2,45	3,00	2,57	1,83	2,50	0,23
(3)	**Alignment of resources**		**3,00**	**2,32**	**2,50**	**2,64**	**2,65**	**0,08**
	2	Organizational and logistical routine for MR-HIFU within center	2,73	2,78	2,71	2,83	2,76	0,00
	28	Availability of centers with HIFU treatment facilities	2,91	2,00	3,14	2,83	2,72	0,25
	29	Availability of a hospital bed for overnight stay	2,64	1,78	1,17	1,83	2,06	0,36
	31	Frequency of time slots at the MRI dedicated for HIFU	3,36	2,38	2,71	3,00	2,91	0,17
	32	Scarcity of MRI resources	2,91	2,00	2,29	1,83	2,34	0,23
	43	Availability of anesthesiologist for MR-HIFU procedures	3,45	3,00	2,43	3,50	3,13	0,25
(4)	**Logistics and workflow**		**2,56**	**2,33**	**2,66**	**2,61**	**2,53**	**0,02**
	4	Referral routine within center	2,36	2,11	3,14	2,83	2,55	0,21
	5	Referral routine to external centers offering MR-HIFU	2,55	2,25	2,57	2,40	2,45	0,02
	30	Collaboration between different health care professionals	3,00	2,89	2,86	3,17	2,97	0,02
	44	Anesthesiological approaches tailored for MR-HIFU	2,27	2,13	2,14	1,83	2,13	0,03
	46	Lack of an established patient workflow (from HIFU-indication to release of the patient)	2,64	2,25	2,57	2,83	2,56	0,06
(5)	**Technical disadvantages**		**2,27**	**2,04**	**2,46**	**2,17**	**2,24**	0,03

	7	MR-HIFU is a lengthy procedure	2,55	2,67	2,71	2,67	2,64	0,00
	8	Instability of interface of MR scanner with HIFU system	1,73	1,86	2,29	1,67	1,87	0,08
	27	Compatibility of HIFU equipment with the MR scanner delay the setup of treatment facilities	1,82	1,63	2,14	1,83	1,84	0,04
	33	Scarcity of HIFU equipment	3,00	2,00	2,71	2,50	2,59	0,18
(6)	**Radiotherapy as first-line therapy**		**2,85**	**2,95**	**3,05**	**2,67**	**2,89**	**0,03**
	7	HIFU is less flexible with respect to different anatomical regions compared to radiotherapy	2,64	2,63	2,86	2,67	2,69	0,01
	26	Competitive logistical advantage of radiotherapy	2,82	3,11	3,00	2,67	2,91	0,04
	47	HIFU treatment procedure complexity compared to radiotherapy	3,09	3,11	3,29	2,67	3,06	0,07
(7)	**Aggregating knowledge & Improving awareness**		**2,65**	**2,61**	**2,61**	**2,58**	**2,62**	**0,00**
	3	Inter-center knowledge exchange	2,27	2,25	2,00	3,00	2,34	0,19
	12	Clear position of MR-HIFU in clinical guidelines	3,09	3,11	3,29	3,33	3,18	0,02
	15	Synergy of incorporating MR-HIFU for other clinical indications and treatment regimes	2,64	2,63	3,00	2,50	2,69	0,05
	39	Users' perception of lack of cost-effectiveness for pain palliation "only"	2,60	2,44	2,14	1,50	2,25	0,24
(8)	**Clinical effectiveness**		**2,50**	**3,19**	**3,19**	**3,33**	**2,99**	**0,14**
	9	Safety profile of MR-HIFU (few or no side effects)	2,36	2,67	3,14	3,17	2,76	0,15
	34	Clinical evidence from randomized clinical trials on the effectiveness of MR-HIFU	2,50	3,56	3,57	3,50	3,22	0,27
	35	Experience/ Observation of positive outcomes after treatment with MR-HIFU	2,64	3,33	2,86	3,33	3,00	0,12
(9)	**Patients' preferences**		**2,55**	**2,94**	**2,79**	**2,67**	**2,73**	**0,03**
	19	Enthusiasm for the non-invasive treatment	2,45	3,00	2,43	2,33	2,58	0,09
	22	Patient preference for an outpatient procedure	2,82	2,33	3,14	2,17	2,64	0,20
	23	Fast recovery after treatment	2,45	2,75	2,86	3,17	2,75	0,09
	36	Superior pain relief compared to radiotherapy	2,45	3,67	2,71	3,00	2,94	0,28
(10)	**Reimbursement**		**2,33**	**3,00**	**3,05**	**1,83**	**2,56**	**0,34**
	10	Reimbursement of MR-HIFU as inpatient procedure	2,73	3,13	2,86	1,67	2,66	0,41
	11	Reimbursement of MR-HIFU as outpatient procedure	1,82	3,00	3,43	2,00	2,50	0,60
	41	Reimbursement to offset the costs of supporting personnel (anesthesia)	2,45	2,88	2,86	1,83	2,53	0,24
(11)	**Cost-effectiveness**		**2,36**	**2,56**	**2,64**	**2,73**	**2,55**	**0,02**
	40	Evidence on cost-effectiveness in relation to standard of care	2,55	3,25	3,00	3,00	2,91	0,09
	49	Reduced costs compared to surgery	2,18	1,88	2,29	2,50	2,19	0,07
(12)	**Hospital costs**		**2,44**	**2,33**	**2,75**	**2,25**	**2,45**	**0,05**
	37	Costs of equipment maintenance	2,45	2,25	2,86	2,00	2,41	0,13
	38	Running costs	2,50	2,75	3,14	2,50	2,71	0,09
	42	High additional costs related to general anesthesia	2,18	2,44	2,00	2,33	2,24	0,04
	48	Costs of initial setup (purchase of equipment, installation, etc.)	2,64	1,88	3,00	2,17	2,44	0,25

Abbreviations: IT: Italy, NL: the Netherlands, DE: Germany, FI: Finland

File S6. Statements Located in the Northeast Quadrant of the Go-Zone

Table S4. Statements located in the northeast quadrant of the Go-Zone (sorted by cluster)

Cluster	Number (%) of statements in the Go-zone	ID	Statement
(2) Physicians' attitude	2(33%)	13	Unfamiliarity/ lack of knowledge among referring physicians with MR-HIFU as a treatment option
		14	Difficult patient recruitment, due to large range in referring medical specialists
(3) Alignment of resources	3(50%)	2	Organizational and logistical routine for MR-HIFU within center
		28	Availability of centers with HIFU treatment facilities
		31	Frequency of time slots at the MRI dedicated for HIFU
(4) Logistics and workflow	3 (60%)	4	Referral routine within center
		30	Collaboration between different health care professionals
		46	Lack of an established patient workflow (from HIFU-indication to release of the patient)
(5) technical disadvantages	1(25%)	33	Scarcity of HIFU equipment
(6) Radiotherapy as first-line therapy	2(67%)	25	HIFU is less flexible with respect to different anatomical regions compared to radiotherapy
		47	HIFU treatment procedure complexity compared to radiotherapy
(7) Aggregating knowledge & Improving Awareness	2 (50%)	12	Clear position of MR-HIFU in clinical guidelines
		15	Synergy of incorporating MR-HIFU for other clinical indications and treatment regimes
(8) clinical effectiveness	3 (100%)	9	Safety profile of MR-HIFU (few or no side effects)
		34	Clinical evidence from randomized clinical trials on the effectiveness of effectiveness MR-HIFU
		35	Experience/ Observation of positive outcomes after treatment with MR-HIFU
(9) Patients' preferences	4 (100%)	19	19 Enthusiasm for the non-invasive treatment
		22	Patient preference for an outpatient procedure preferences
		23	Fast recovery after treatment
		36	Superior pain relief compared to radiotherapy
(10) Reimbursement	1 (33%)	11	Reimbursement of MR-HIFU as outpatient procedure
(11) Cost-effectiveness	1 (50%)	40	Evidence on cost-effectiveness in relation to standard of care
